# Advanced lung organoids and lung-on-a-chip for cancer research and drug evaluation: a review

**DOI:** 10.3389/fbioe.2023.1299033

**Published:** 2023-11-07

**Authors:** Leqing Zhu, Jianhua Zhang, Quanwei Guo, Jun Kuang, Dongfang Li, Mengxi Wu, Yijun Mo, Tao Zhang, Xinghua Gao, Jianfeng Tan

**Affiliations:** ^1^ Department of Thoracic Surgery, Shenzhen Hospital, Southern Medical University, Shenzhen, China; ^2^ Shenzhen Clinical Medical College, Southern Medical University, Shenzhen, China; ^3^ Materials Genome Institute, Shanghai University, Shanghai, China

**Keywords:** 3D culture system, lung organoid, lung-on-a-chip, lung cancer, drug evaluation

## Abstract

Lung cancer has become the primary cause of cancer-related deaths because of its high recurrence rate, ability to metastasise easily, and propensity to develop drug resistance. The wide-ranging heterogeneity of lung cancer subtypes increases the complexity of developing effective therapeutic interventions. Therefore, personalised diagnostic and treatment strategies are required to guide clinical practice. The advent of innovative three-dimensional (3D) culture systems such as organoid and organ-on-a-chip models provides opportunities to address these challenges and revolutionise lung cancer research and drug evaluation. In this review, we introduce the advancements in lung-related 3D culture systems, with a particular focus on lung organoids and lung-on-a-chip, and their latest contributions to lung cancer research and drug evaluation. These developments include various aspects, from authentic simulations and mechanistic enquiries into lung cancer to assessing chemotherapeutic agents and targeted therapeutic interventions. The new 3D culture system can mimic the pathological and physiological microenvironment of the lung, enabling it to supplement or replace existing two-dimensional culture models and animal experimental models and realize the potential for personalised lung cancer treatment.

## 1 Introduction

Lung cancer is a common cancer worldwide and the leading cause of cancer-related deaths. In 2020 alone, over 2.2 million new cases and nearly 1.8 million deaths were attributed to lung cancer (Sung et al., 2021). In particular, lung cancer is complex and diverse with different subtypes, including small cell lung cancer (SCLC) and non-small cell lung cancer (NSCLC), which can also be divided into adenocarcinoma, squamous cell carcinoma (SCC), and large cell neuroendocrine carcinoma (LCNEC). Advancements in comprehensive treatments, including surgery, radiotherapy, chemotherapy, gene-targeted therapy, and immunotherapy, have improved therapeutic outcomes and prolonged survival in patients with lung cancer. However, various challenges remain, such as tumour recurrence, invasive metastasis, and drug resistance. Therefore, personalised diagnostic and therapeutic strategies are required, and more accurate lung cancer models must be developed.

Platforms for studying lung cancer and drug evaluation can be divided into *in vitro* and *in vivo* models, including two-dimensional (2D) cell culture, animal models, and three-dimensional (3D) culture systems such as organoids and organ-on-a-chip. *In vitro* 2D cell cultures are typically used models for studying tissue pathophysiology and drug response (Gazdar et al., 2010); however, owing to the limitations of cell culture conditions, they cannot fully simulate the specificity of human tissue structure, mechanics, and function (Sugaya et al., 2002). Animal models have also been used to simulate human physiological and pathological microenvironments for disease research, preclinical drug development, and screening. However, because of the inevitable differences in physiological structure, tissue and organ function, life maintenance, and other aspects between animals and humans, animal models cannot accurately simulate the physiological and pathological environment of the human body (Hartung, 2009; Snoeck, 2015); thus, experimental results from animals cannot be used to predict human drug reactions. Moreover, recent biological therapies using monoclonal antibodies and gene vectors require experimental models that include specific human target molecules or conformations that animal models cannot provide (Ingber, 2022). In addition, animal experiments require longer model manufacturing times, lower stability, and ethical considerations (Perelson and Ribeiro, 2018). Therefore, owing to the limitations of 2D cell cultures and animal models, efficient new approaches are required to develop advanced 3D models for disease modelling, drug development, and screening. At the same time, compared to general 3D models, the construction of lung or lung tumour 3D models is more challenging. Because the lungs, as the respiratory organs of the human body, have a special series of biological features and unique respiratory membranes. Its features include biophysical and biochemical factors, such as special gases, fluids, soluble cytokines, air-liquid interfaces and respiratory movements, which are clearly different from other 3D models. Therefore, the study of advanced 3D lung culture system models, especially lung organoids and lung-on-a-chip, requires the integration of multiple fields such as materials science, tissue engineering, and biomedicine.

**FIGURE 1:**
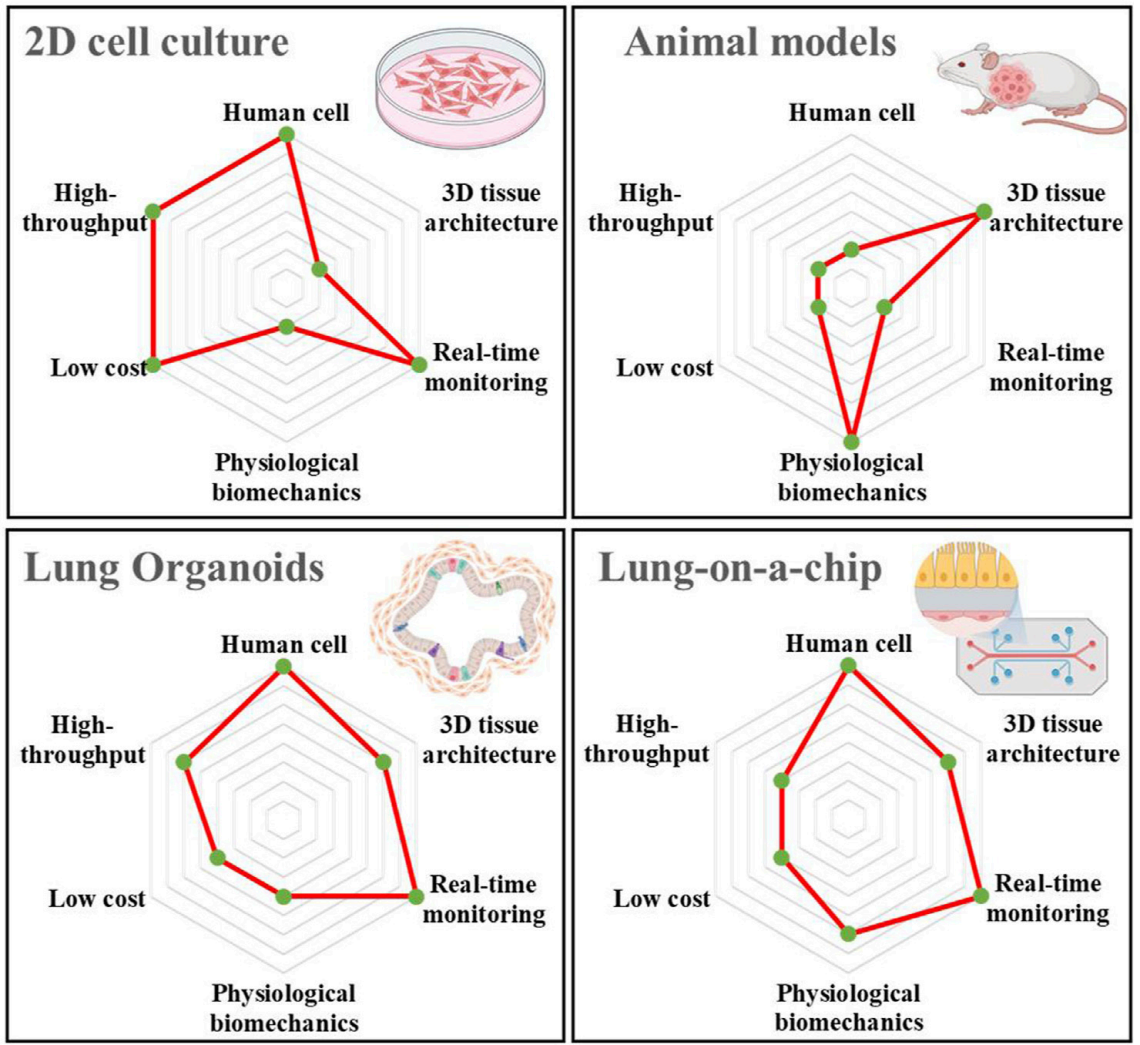
Comparison of the relevant characteristics and advantages of the four current lung cancer models, including 2D cell culture, animal models, lung organoid, and lung-on-a-chip. The illustrations are created with BioRender.com.

In recent years, with the continuous progress in microfabrication and tissue engineering technologies, the construction of advanced 3D culture systems has enabled the establishment of biomimetic 3D human tissue or organ models, which may solve the problems prevalent in existing 2D cell culture and animal models (Tan et al., 2022; Tian et al., 2023). Moreover, these models can more accurately simulate lung cancer and evaluate drug efficacy (Li et al., 2023). Among them, organoids and organ-on-a-chip systems belong to advanced 3D culture systems. Organoids are complex multicellular clusters generated during the *in vitro* culture of stem cells related to a specific organ. The 3D microenvironment of the model framework comprises both a simulation of the corresponding disease environment to form a complex tissue structure and a simplified version of the organ. In contrast, organ-on-a-chip is a biomimetic device mimicking the main functions of human organs using microfluidic chips. These devices have microscale gas or fluid channels that can simulate the tissue microenvironment and blood circulation system, constructing certain tissue–tissue interfaces and organ–organ interfaces, thereby simulating microenvironments, complex structures, and biophysical factors of human organs. Consequently, they can address the limitations in animal experiments, such as species differences, long experimental cycles, high costs, and ethical concerns (Perelson and Ribeiro, 2018; Li et al., 2023). Figure 1 shows a comparison between the traditional 2D cell cultures, animal models, and 3D culture systems regarding human cells, tissue architecture, real-time monitoring, physiological biomechanics, low cost, and high throughput. Among them, lung organoids and lung-on-a-chip have significant advantages in human cell culture and real-time monitoring compared to animal models. In particular, compared with 2D cell cultures, they have significant advantages regarding tissue architecture and physiological biomechanics. More importantly, the organ-on-a-chip can shorten the research process by enhancing the stability of the culture system for drug development research, which in turn improves the performance of drug efficacy evaluation (Ingber, 2022). Studies have shown that the organ-on-a-chip significantly reduces R&D costs by approximately 25% of the total process, and can also bring additional benefits (Franzen et al., 2019). Both are acceptable regarding cost and throughput, providing a new strategy for solving the problems in current 2D cell cultures and animal models. In this review, we summarise novel lung-related 3D culture systems, including lung organoids and lung-on-a-chip. Moreover, we focus on the latest progress in lung cancer research and drug evaluation, including lung cancer simulation and mechanism research, chemotherapeutic drugs, and targeted drug evaluation. Novel 3D culture systems can mimic the pathological or physiological microenvironment of the lung, enabling it to supplement or replace existing 2D culture and animal experimental models, indicating its potential for personalised lung cancer treatment.

## 2 Lung organoids

Lung organoids can be developed using patient-derived tumour cells (PDCCs) or human embryonic stem cells (ESCs) along with key cytokines critical for lung development. These organoids aim to replicate the growth and differentiation of the lung *in vitro*, enabling a more precise examination of tumour growth mechanisms and evaluation of new anti-cancer medications (Shi et al., 2020). Sources of lung organoid cells primarily comprise lung stem cells, lung mesenchymal stromal cells (MSCs) with differentiation potential, and PDCCs. Moreover, Pluripotent stem cells (PSCs) can be obtained from ESCs and induced pluripotent stem cells (iPSCs). Lung organoids were first developed in 1981 using ESCs (Evans and Kaufman, 1981). In 1987, Zimmermann et al. cultured mouse foetal lung cells, resulting in a lung organoid with alveolar-like cavities and a basal lamina at the interface between the medium and air (Zimmermann, 1987). In 2010, Franzdottir et al. co-cultivated human bronchial cells with human umbilical vein endothelial cells (HUVECs) to form bronchioalveolar-like structures (Franzdottir et al., 2010). However, this 3D model suffers from the inability to fully represent the histologic differences that exist within the proximal and distal lung and airway epithelium, as well as the dependence on lung stem cells. In 2020, Meyer-Berg et al. created lung bud organoids (LBOs) for patients with certain lung genes using human ESCs and recombinant adeno-associated viruses (rAAV) (Meyer-Berg et al., 2020). In 2019, Miller et al. differentiated human pluripotent stem cells (hPSCs) into human lung bronchial and fine bronchial-like organoids in 2019 (Miller et al., 2019). Moreover, iPSCs were prepared by Takahashi and Yamanaka (2006), representing a milestone event and leading to new opportunities for stem cell research. In contrast to embryonic stem cells (ESCs), which are derived from ESCs, iPSCs can be generated by somatic gene reprogramming. This has expanded the sources of stem cells in lung organoids. In 2020, Leibel et al. produced a 3D multicellular organoid by inducing differentiation of human ESCs and iPSCs, including epithelial cells, MSCs, ciliated cells, and alveolar macrophages (Leibel et al., 2020). The process of organogenesis, which involves multiple cell screens and timing of cytokine additions, which can be combined with automated modes to obtain higher yields with fewer treatments.

**FIGURE 2:**
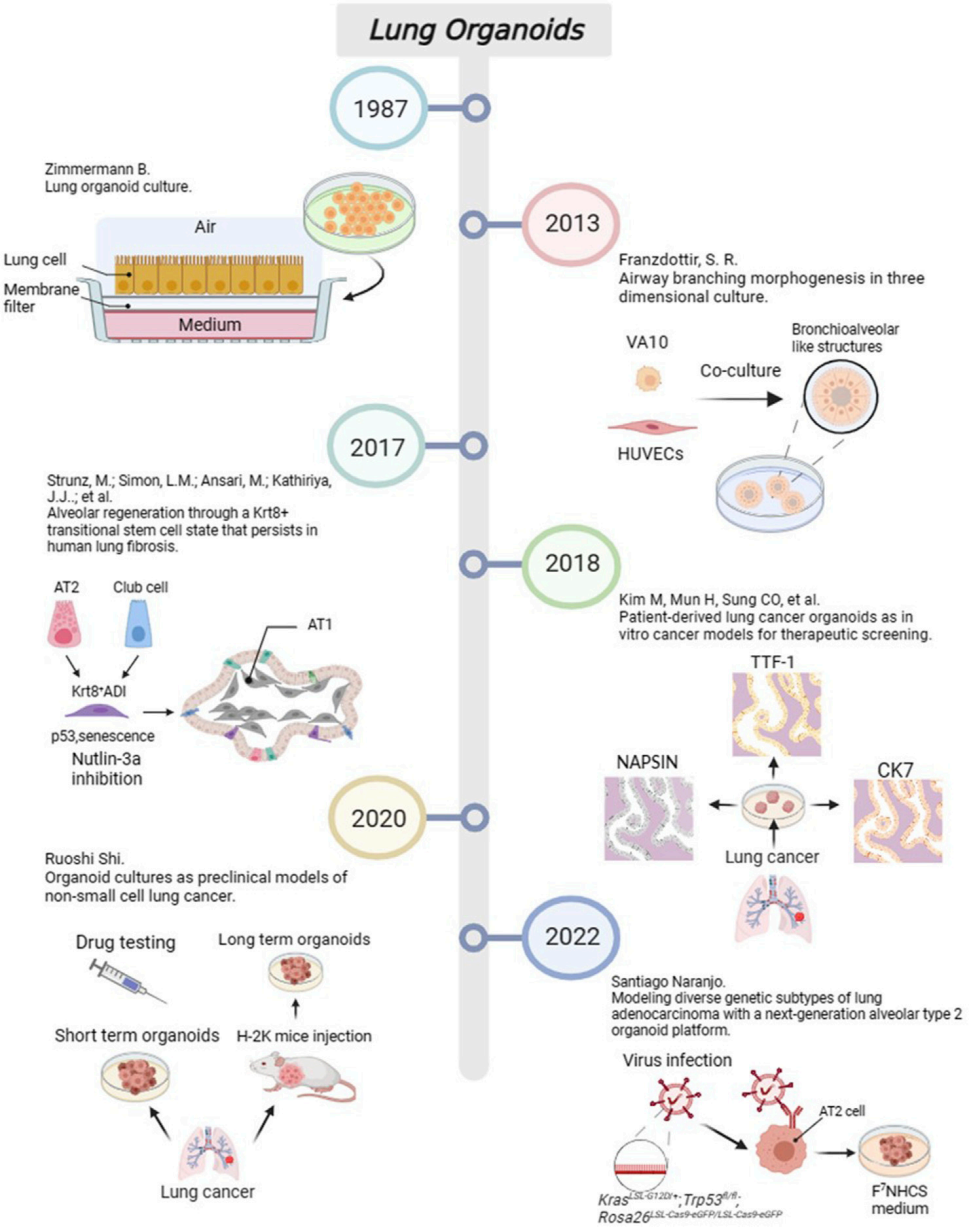
The progress of important events in lung organoids. Created with BioRender.com.

From the perspective of disease models, researchers have primarily focused on constructing organoid models of and performing pharmacological research on five major diseases, including asthma, chronic obstructive pulmonary disease (COPD), fibrotic diseases, infections, and lung tumours. For example, Notch2 has been identified as a key regulatory factor promoting goblet epithelial hyperplasia (GCM) in the bronchial airway, which is also a leading cause of asthma and COPD. Therefore, simulation of Notch2 expression in bronchial cells may explain the mechanism of disease occurrence. In 2015, Danahay introduced an organoid model with alveolar epithelial cells that could simulate the proliferation of goblet cells and mucus increase in asthma (Danahay et al., 2015). It fully reduced the 3D structure of the inflammatory changes in multilayer. including basal cell progenitors, goblet cells, and ciliated cells. In addition, Rao et al. used single-cell cloning technology to culture lung tissues from patients with COPD and studied the mechanisms of inflammation, fibrosis, excessive mucus secretion, and metaplastic epithelial lesions in 2020 (Rao et al., 2020). Lung organoid models have also been used to study fibrotic diseases such as idiopathic pulmonary fibrosis (IPF). In 2017, Surolia et al. cultured resected tissues from patients with IPF to form 3D organoids (Li et al., 2017). Alternatively, Wilkinson et al. established an IPF-style model of progressive scar formation using embryonic lung fibroblasts or induced pluripotent stem cell-derived mesenchymal cells treated with transforming growth factor-β (TGF-β) (Wilkinson et al., 2017). Moreover, organoid models can be used to study various types of infection models by simulations, such as infection caused by *Mycobacterium* (Iakobachvili et al., 2022) and *Streptococcus* (Sempere et al., 2022). Virus-infected lung organoid models have recently attracted increasing attention. In 2019, Sachs et al. infected organoids containing the small airway epithelium with a respiratory syncytial virus to explore the effect of viruses on airway remodelling (Sachs et al., 2019). The outbreak of SARS-CoV-2 in 2020 resulted in several studies examining lung organoids infected with COVID-19. Han et al. used a lung organoid model of hPSCs to investigate the strong induction effects of SARS-CoV-2 infection on chemokines (Han et al., 2021). In recent years, with the improvement of the manufacturing technology of the models and further understanding of the growth factors required for tumour cells, the lung cancer organoid (LCO) model has been developed from foundation to maturity. Generally, the process of constructing an LCO involves the following steps: selecting appropriate samples from sources such as surgical resection specimens, pleural effusion, or sputum from patients, and seeding the cells into plates containing growth factors after washing, incubation, dissociation, labelling, magnetic separation, and other steps. Finally, the function and value of LCO were assessed by measuring the content of tumour markers and verifying the purity of the cells in the tumour organ. In 2018, Clevers et al. developed an early LCO model (Dijkstra et al., 2018), and in 2019, Kim et al. defined 80 cases of LCOs containing five types of lung cancer (Kim et al., 2019). The milestone events in the development of lung organoids in recent years is shown in Figure 2.

## 3 Lung-on-a-chip

Organ-on-a-chip is a biomimetic device with microscale gas or fluid channels that can simulate tissue and blood circulation systems. Organs can build tissue-tissue interfaces and organ-organ interfaces, simulating the microenvironment, complex structure and biophysical factors of human organs. It is more accurate and realistic compared to the two-dimensional model, and it can solve the shortcomings of animal experiments, such as species differences, long cycle time, high cost, animal ethics and other issues (Perelson and Ribeiro, 2018; Li et al., 2023). This device is used as a model for evaluating drug toxicity and efficacy by constructing certain tissue-tissue interfaces and organ–organ interfaces to simulate the microenvironment, complex structure, and biophysical factors of human organs. As one of the earliest organ-on-a-chip systems proposed and developed, the lung-on-a-chip can be applied not only to model development and drug evaluation of pulmonary oedema, pulmonary thrombosis, viral infection, and other diseases (Takahashi and Yamanaka, 2006; Danahay et al., 2015; Leibel et al., 2020; Rao et al., 2020) but also to model building and drug evaluation of lung tumour (Li et al., 2017; Wilkinson et al., 2017).

Moreover, lung-on-a-chip can be used to culture normal human alveolar epithelial cells, HUVECs (Huh et al., 2010), respiratory muscle tissue cells (Humayun et al., 2018), primary lung cancer cells (Xu et al., 2013), a single lung cancer cell line (Li et al., 2020), lung cancer fibroblast cell lines (Yang et al., 2018), and additional types of cellular components of the mixture on the microchip. Its main structure comprises upper and lower microfluidic channels. The upper microchannels can be used to culture alveolar epithelial cells, forming air–liquid interface to simulate alveoli by applying oxygen. The lower microchannels can be used to culture HUVECs and continuously perfuse culture fluid to simulate capillary channels and fluid shear stress. The middle of upper and lower microchannels sandwich a porous membrane to simulate alveolar septa. Side chambers exist on both sides of the lung-on-a-chip connected to two vacuum pumps; the porous film is regularly stretched by changing the pressure of the side chambers, which simulates the respiratory motion of the lungs (Tan et al., 2023). Cellular components are placed into their respective channels and cultured in a continuously replenished 3D medium such that after a certain period, the cells form functional tissue units (Xu et al., 2013). The materials of lung-on-a-chip are the basis for their fabrication and application. Materials typically used include polydimethylsiloxane (PDMS), paper, poly lactic-co-glycolic acid (PLGA), and extracellular matrix (ECM). In 2010, Huh et al. established an original lung-on-a-chip model, which consisted of alveolar epithelial cells and HUVECs, reproducing the alveolar-capillary interface and the air-blood barrier (Huh et al., 2010). In 2014, Sellgren et al. added lung fibroblasts to Huh’s lung-on-a-chip (Sellgren et al., 2014). In the same year, McCain et al. created a lung organ-on-a-chip model containing human respiratory musculature (Mccain et al., 2014). The main advantages of PDMS are non-toxic, light-transmitting, chemically inert, easy to process, and inexpensive, being widely used in the fabrication of lung microarrays, and its main disadvantage is relatively hydrophobic (Taylor et al., 2005), which often requires the modification of a layer of biocompatible extracellular matrix or hydrogel material (Park et al., 2006). Another drawback is the high uptake rate of hydrophobic drugs (Shirure and George, 2017). In 2015, Stucki et al. used photolithography to create a lung-on-a-chip containing a stretched alveolar barrier to mimic the diaphragm during *in-vivo* respiratory movements (Stucki et al., 2015). In 2016, Ziaie et al. used parchment paper treated with a laser as a semi-permeable membrane to construct an air-liquid interface to culture monolayer lung epithelial cells (Rahimi et al., 2016). There are drawbacks to this type of parchment paper, one of which is that it requires laser processing in order to form hydrophilic regions, and the other is that prolonged *in vitro* incubation can lead to a weakening of the wet and dry tensile strength, and may even disrupt the semipermeable membrane. In 2018, Hu et al. developed a thermoplastic model of a lung-on-a-chip that recapitulated the lung airway microenvironment and the interactions between smooth muscle cells, epithelial cells, and the supporting extracellular matrix (Humayun et al., 2018). In the same year, Gao et al. used PLGA nanofiber membranes for the first time with extremely thin thickness (approximately 3 μm) and improved biocompatibility and porosity as an intermediate porous film material (Yang et al., 2018). However, biophysical factors such as fluid shear and tensile stress cannot be simulated due to the lack of strength of the PLGA material. In 2019, Park et al. used 3D cellular bioprinting, mixed polycaprolactone, lung fibroblast bioink, endothelial cell bioink, and PDMS to construct a lung-on-a-chip platform (Park et al., 2018). Finally, in 2021, Guenat et al. used collagen and elastin to design a lung-on-a-chip with biogenic, stretchable, and degradable membranes mimicking arrays of tiny alveoli *in vivo* dimensions (Zamprogno et al., 2021). ECM membrane is better than PDMS in many ways, not absorbing hydrophobic drugs, biogenic, stretchable, and degradable, can be prepared very thin membranes (around 4 μm), but its degree of toughness is not sufficient.

**FIGURE 3:**
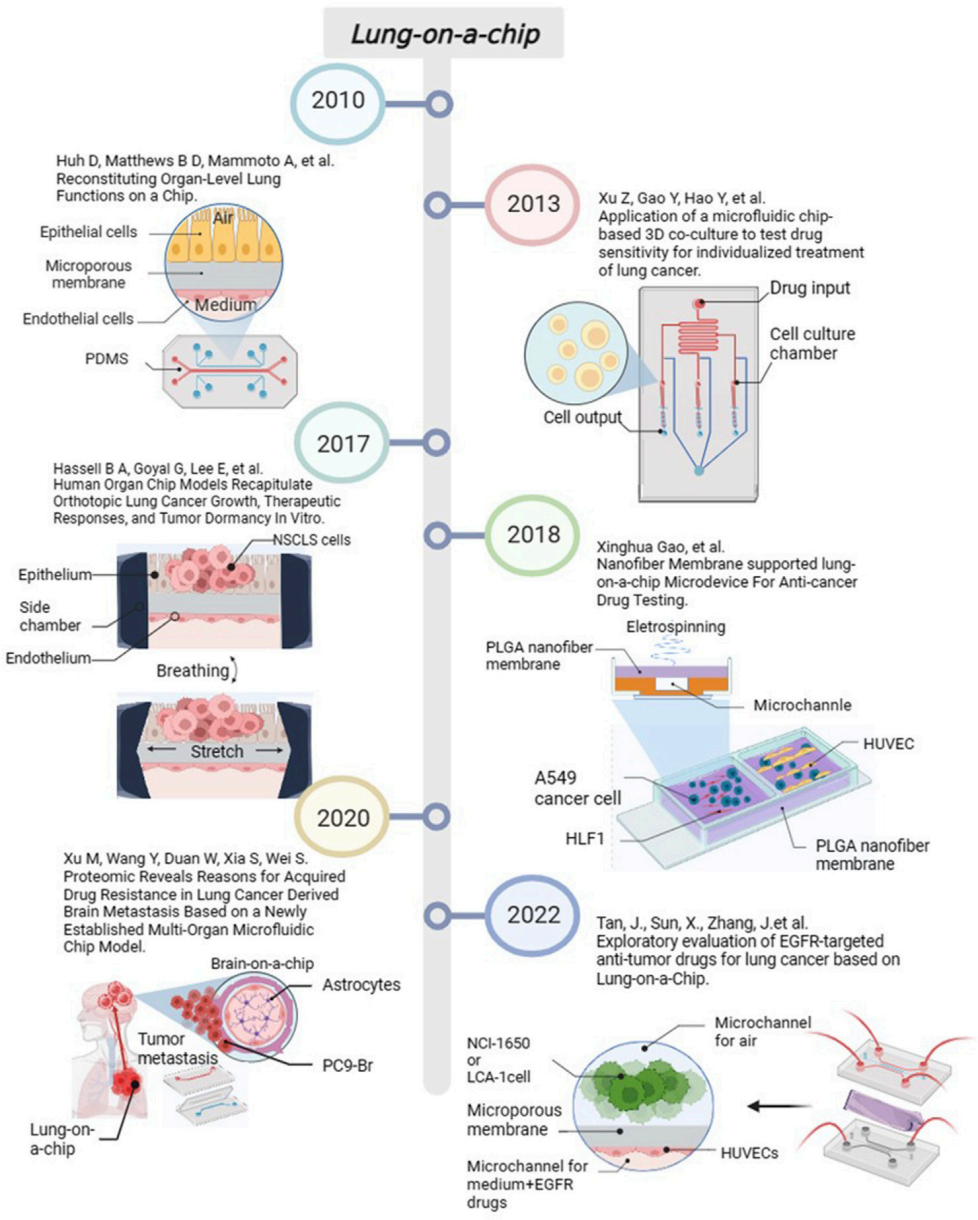
The progress of important events in lung-on-a-chip. Created with BioRender.com.

Lung-on-a-chip is currently used for disease simulation in inflammation, pathogen infection, and lung tumors. In 2016, Benam et al. designed a small bronchial chip for simulating inflammatory responses in the small airways at the sub-organ level (Benam et al., 2016). After that, researchers recognized that pathogens can affect the lung epithelial structure and function of lung-on-a-chip and gradually began to study models containing host-pathogenic microorganisms. These studied included lung-on-a-chip models containing *Mycobacterium tuberculosis* (Thacker et al., 2020), *Staphylococcus aureus* and influenza A virus co-infected pneumonia (Deinhardt-Emmer et al., 2020), rhinovirus (Nawroth et al., 2020), and virus analog poly (I: C) and SARS-CoV-2 pseudovirus (Cao et al., 2022). In addition, constructing NSCLC models based on lung-on-a-chip has become a current hotspot. Researchers has used NSCLC models to study the development, metastasis, and drug resistance of lung cancer. We have also carried out some work. In 2022, we used NCI-H1650 cells, the primary human NSCLC, and HUVECs to construct a lung-on-a-chip platform for evaluating the response of lung cancer cells to epidermal growth factor receptor (EGFR)-targeted drugs (Tan et al., 2022). The progress of important events in lung-on-a-chip in recent years is summarised in Figure 3.

## 4 Applications of lung organoids and lung-on-a-chip

**FIGURE 4:**
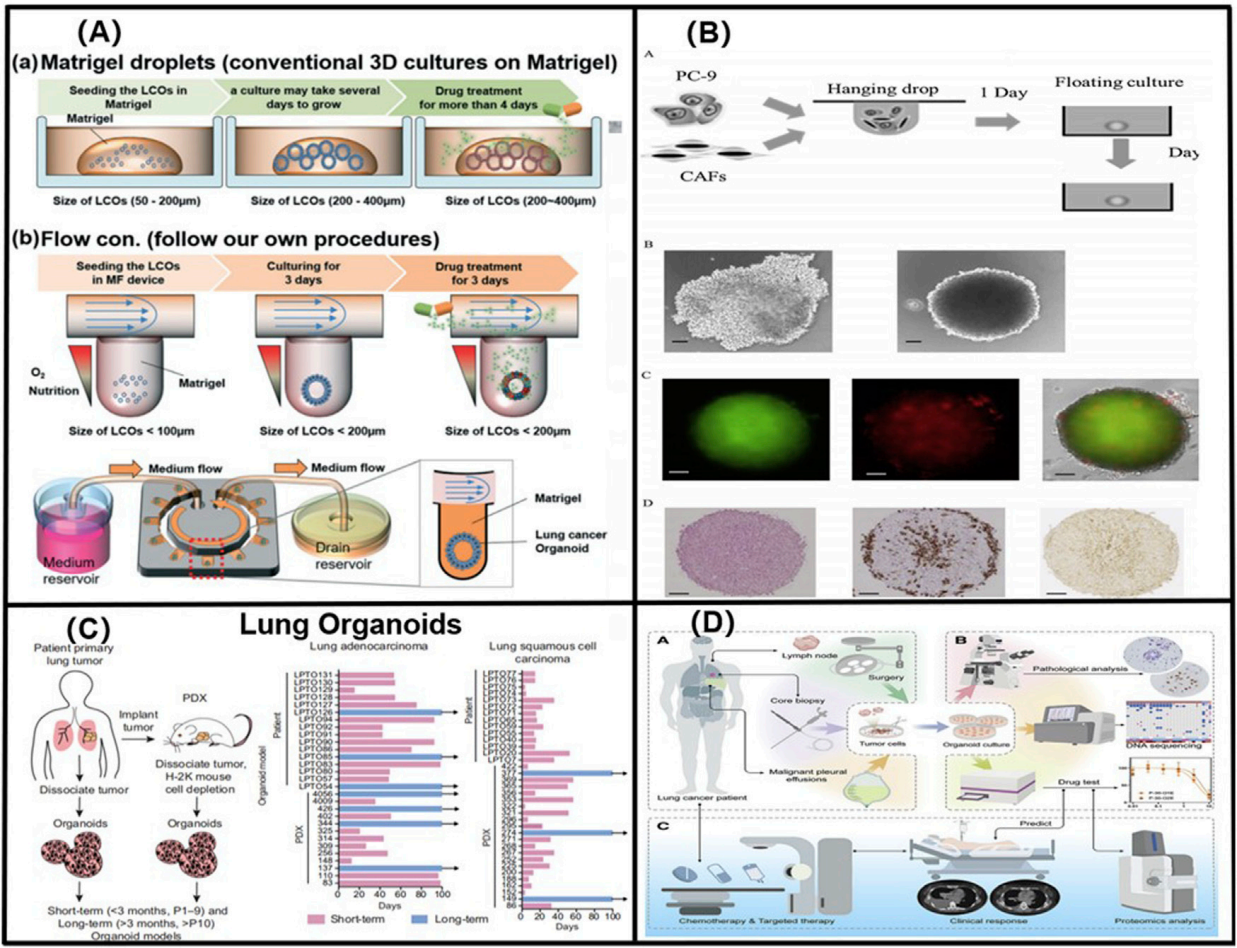
**(A)** 3D lung cancer organoids in a size-controllable manner and demonstrates for the production of lung cancer organoids from patients with small-cell lung cancer. Adapted from Jung et al. (2019). Copyright 2019, Royal Society of Chemistry. **(B)** Using genetic engineering techniques to obtain LCOs with specific Kras-, Trp53-deficient, and Eml53-Alk mutations, which significantly accelerated the study of the lung cancer genetic mechanisms. Adapted from Nakamura et al. (2019). Copyright 2019, Elsevier B.V. **(C)** By constructing long-term (greater than 3 months, more than 10 generations), and short-term (less than 3 months, less than 10 generations) NSCLC organoid models, Shi found that cancer organoids with breast cancer 2 gene, EGFR, and EGFR- and EGFR-mutation/MSC-epithelial-transformation (MET)-amplified mutations responded favourably to lapatinib, erlotinib, and crizotinib, respectively. Adapted from Shi et al. (2020). Copyright 2020, American Association for Cancer Research. **(D)** A LCO-based drug susceptibility test (LCO-DST) of osimertinib, paclitaxel, pemetrexed, carboplatin, etoposide, and cisplatin. Adapted from Wang et al. (2023). Copyright 2023, Cell Press.

**FIGURE 5:**
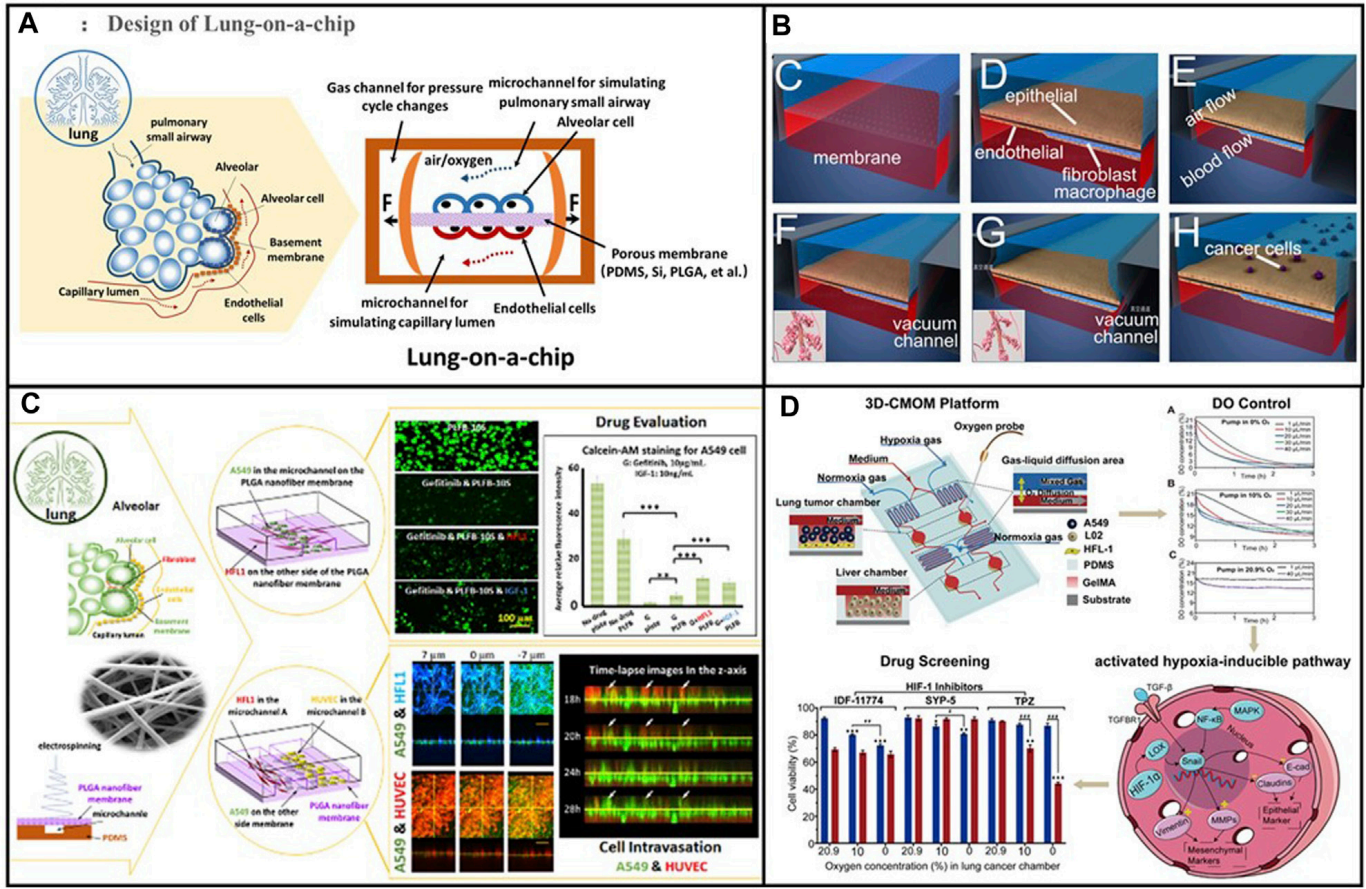
**(A)** Schematic diagram of the alveolar structure. Its main structure comprises upper and lower microfluidic channels. Adapted from Tan et al. (2023). Copyright 2023, Elsevier B.V. **(B)** A multi-organ microarray can further be used to assess the mechanism of epithelial–mesenchymal transition (EMT) in lung cancer cells invading distant tissues and organs, such as the brain, bone, and liver. Adapted from Xu et al. (2016). Copyright 2016, American Chemical Society. **(C)** Co-cultured lung-on-a-chip models of A549 tumour cells and HFL1 to explore the effects of HFL1 on tumour cell metastasis and drug resistance. Adapted from Yang et al. (2018). Copyright 2018, Royal Society of Chemistry. **(D)** The three-dimensional-culture multiorgan microfluidic (3D-CMOM) platform for the cancer treatment effects of HIF-1α inhibitors (tirapazamine, SYP-5, and IDF-11774). Adapted from Zheng et al. (2021). Copyright 2021, American Chemical Society.

To further elucidate the advantages of advanced lung organoids and lung-on-a-chip over traditional systems, some representative images of lung organoids and lung-on-a-chip in Figure 4 and Figure 5 highlight the unique features of 3D systems that are not feasible in animal models or 2D cell cultures. Based on the above lung organoids and lung-on-a-chip, we categorised the principal lung organoids and lung-on-a-chip based on their various features to simulate lung cancer *in situ* and metastatic lung cancer, study the mechanisms of lung cancer development, and evaluate drug treatments, as shown in Tables 1, 2.

**TABLE 1:** Lung cancer research utilizing lung organoid models.

Organoids	Cell types	Features	Mechanism	Drug testing	References
LCOs	Adenocarcinoma	Derived from primary lung cancer tissues and paired non-neoplastic airway tissues	—	Olaparib	Kim et al. (2019)
	SCC	Own a greater long-term expansion potential		Erlotinib	
	ASC	Maintain histological and genetic characteristics		Crizotinib	
	LCNEC				
	SCLC				
LCOs	Cells derived from SCLC patients	A amicrophysiological system	—	Cisplatin	Jung et al. (2019)
		Enabled LCO culturing		Etoposide	
		Finished drug sensitivity tests			
AOs	NSCLC	Human airway organoids from broncho-alveolar	The dramatic causes of RSV infection and the neutrophil–epithelium interaction in epithelial airway organoids	Paclitaxel	Sachs et al. (2019)
	CF cell	Modeled RSV infections		Methotrexate	
		Presented the direct effects of the viral protein NS2		Crizotinib cisplatin	
LUAD organoids	PC-9 cancer cell	High cultivation success rate	Examined the effect of podoplanin (+) CAFs on the proliferation of cancer cells in the hybrid cancer organoids	—	Nakamura et al. (2019)
	CAFs	Co-cultured the generation of hybrid cancer organoids			
		Contains both cancer cells and podoplanin (+) CAFs			
LUAD and LUSC organoids	ASC	Cultured short-term and long-term organoids	—	Trametinib	Shi et al. (2020)
		Preserve the mutation and copy number landscape		Selumetinib	
		Combination therapy in NSCLC organoids		BGJ398+	
				Trametinib	
				Combination	
LCOs	NCI-H460	Cultured the cisplatin-resistant lung cancer organoids	HF induced G0/G1 phase arrest and apoptosis	HF, cisplatin	Li et al. (2021)
	NCI-H1299	Evaluated the combination of cisplatin and HF	HF affected PI3K/AKT and MAPK signaling pathways		
AT2 organoid	AT2 cell prominent cell of origin for LUAD	Organoid models of KRAS, BRAF, and ALK mutant	Chromosomal inversion leads to an oncogenic fusion between Eml4 and Alk using CRISPR/Cas9 technologies	—	Naranjo et al. (2022)
		Cultured cell lines in F7 NHCS and growth factor-depleted media			
		Tracked tumor-immune interactions in transplanted antigen-expressing organoids			
LCOs	Adenocarcinoma	Provided a personalized platform for the treatment of patients with unknown lung cancer	Osimertinib, BLU-667, and the combination of three groups showed great differences in proteomic profiles which indicated the mechanisms of drug resistance	Osimertinib	Wang et al. (2023)
	SCC	Tested both targeted therapy and chemotherapy		Nabpaclitaxel	
	SCLC			Pemetrexed	
	ASC pulmonary sarcomatous carcinoma			Carboplatin etoposide cisplatin	
PDOs	PDCC isolated from the malignant pleural effusion	Provided a model system that enables intimate investigation of the behaviors of cancer cells in the body	PDSs contributed to the enhancement of TGF-β to induce EMT, while PDOs attenuated it	—	Surina et al. (2023)
		Compared the PDSs and PDO in both the interaction to the immune systems and to the stroma			
LCOs	LUAD	Used genetic engineering to make the induction of Wnt independency by EGFR/Ras alterations	Wnt-dependent and –independent phenotypes in lung adenocarcinoma are defined by NKX2-1 expression	Porcupine inhibitor (C59)	Ebisudani et al. (2023)
	LUSC	Introduced sgRNA targeting NKX2-1 into three lines of NKX2-1+ WRi LUAD organoids	Loss of the NKX2-1 sensitizes human lung adenocarcinomas to Wnt-targeting therapy		
	SCLC	Used targeting Wnt signaling for the treatment of LUAD			
	LCNEC				

NSCLC, non-small cell lung cancer; SCLC, small cell lung cancer; SCC, squamous cell carcinoma; ASCs, adenosquamous carcinoma cells; LCNEC, large cell neuroendocrine carcinoma; LCO, lung cancer organoid; AOs, airway organoids; RSV, respiratory syncytial virus; CF, cystic fibrosis; LUAD, lung adenocarcinoma; CAFs, cancer-associated fibroblasts; LUSC, lung squamous cell carcinoma; HF, halofuginone; AT2, alveolar type 2; PDOs, patient-derived oganoids.

**TABLE 2:** Lung cancer research utilizing Lung-on-a-chip models.

Construction materials	Cell types	Features	Mechanism	Drug testing	References
ECM-coated membrane	Human adenocarcinoma cells, H1975	Monitored the growth and invasion of the orthotopic lung cancer	The changes in signaling through EGFR- and MET protein kinase may cause resistance to therapy	Rociletinib	Hassell et al. (2017)
		Analysis the effects of mechanical breathing on cancer behaviors			
		Analysis EGHR-TKI therapeutic responses to physical modulation			
PLGA electrospinning nanofiber membrane	Human NSCLS cell A549, HFL1, HUVECs	Co-culture of A549 cells and HFL1 cells in a microchip	IGF-1 secreted by HFL1 cells could maintain the tumor cells by activating the PI3K/Akt signal pathway, resulting in decreased sensitivity of the tumor cells to drugs	Gefitinib	Yang et al. (2018)
		Prepared four kinds of PLGA nanofiber membranes			
		Evaluation of EGFR-targeted anti-tumor drug			
PLGA and PDMS membrane	Adenocarcinoma cell lines NCI-H460, Large cell carcinoma NCI-H1650	A novel poly PLGA nanofiber/PDMS microporous composite membrane	The mechanism of the resistance of NCI-H460 cells to gefitinib under normoxic and hypoxic conditions	Gefitinib	Li et al. (2020)
		EGFR-Targeted drug evaluation under normoxia and hypoxia conditions			
ECM membrane	Human NSCLS cell A549, HUVECs	Delivered miR-497-loaded exosomes to co-cultured A549 cells with HUVECs in a microfluidic 3D lung cancer model	MiRNA-497 can suppress the tumor growth and the expression of their associated genes, like YAP1, HDGF, CCNE1, and VEGF-A, and decrease the VEGF-A-mediated angiogenic sprouting	MiRNA-497	Jeong et al. (2020)
		Investigated the anti-tumor and anti-angiogenic effects of miRNAs on NSCLCs			
Hydrogel	Human NSCLS cell A549 and hAM-MSCs	Developed A549 and hAM-MSCs cells spheroid in a compartmentalized microfluidic device	Reduced the viability and the proliferative capacity of cancer by expressing cell proliferation marker Ki-67	A novel tryptophan-rich peptide P1, ACP	Dhiman et al. (2020)
		Tested cytotoxic effects of ACP on tumor spheroids			
PDMS polydimethylsiloxane membrane	PC9-Br cell line	Established a model of lung cancer brain metastasis based on a newly microfluidic multi-organ chip	GSH metabolism and the overexpression of various GSH metabolism-related enzymes (GPX4, RRM2, GCLC, GPX1, GSTM4, GSTM1). ALDH1A1, ALDH3A1 upregulated in BM	Cisplatin	Xu et al. (2020)
	HBE hPMEC	Revealed the protein expression profile of PC9-Br to drug resistance		Carboplatin	
	HFL1	Compared the different protein expression profile of PC9-Br with parental PC9		Pemetrexed	
	THP-1			Gefitinib	
				AZD3759	
PDMS	A549 cell	Established an organ-level lung cancer and liver linkage model	HIF-1α pathway elevates EMT transcription factors (Snail 1 and Snail 2)	HIF-1α inhibitors (tirapazamine, SYP-5, and IDF-11774)	Zheng et al. (2021)
GelMA	HIF-1 cell line	Explored the gene expression of cancer related markers under the normoxic and hypoxic conditions			
PMMA					
PDMS	A-549 cell	A novel microfluidic chip with a dynamic environment and strategically placed U-shaped wells	—	Paclitaxel	Sankar et al. (2021)
		Combinatory drug treatment in dynamic and static culture		Vinorelbine	
				Etoposide	
PC membrane	NCI-H1650	Developed a microporous membrane lung chip based on 3D printing manufacturing technology	—	Gefitinib	Tan et al. (2022)
	Primary human lung cancer cells	Tested the effects of different EGFR-targeted drugs on NCI-H650 cells and primary lung cancer cells in 2D well plates and 3D lung chips		Afatinib	
	HUVECs			Osimertinib	

ECM-coated membrane, porous extracellular matrix-coated membrane; PLGA, poly lactic-co-glycolic acid; PDMS, nanofiber/polydimethylsiloxane; HBE, human bronchial epithelial cells; HFL1, human lung fibroblasts; THP-1, human mononuclear cells; hPMEC, human pulmonary microvascular endothelial cells; ACP, anticancer peptide; hAM-MSCs, human amniotic membrane mesenchymal stem cells; BM, brain metastases; GelMA, gelatin methacryloyl; PMMA, poly methyl methacrylate; PC, polycarbonate.

### 4.1 Lung cancer modelling via lung organoids and lung-on-a-chip

#### 4.1.1 Lung cancer *in situ*

The most important role of advanced lung organoids and lung-on-a-chip in developing lung cancer models to simulate lung cancer *in situ* is to maintain the tumour tissue characteristics. In 2019, Kim et al. isolated tumour cells from patients to create organoids replicating various carcinoma types, including SCCs, adenocarcinoma, adenosquamous carcinoma cells (ASCs), SCLC, and LCNEC (Kim et al., 2019). Subsequently, in 2020, Shi improved Clevers’ approach to achieve high success rates in developing short- and long-term organoid models of NSCLS (Shi et al., 2020). Due to the limitations of their use of primary cancer cells, these LCOs are unable to mimic the diversity of genetic alterations. Therefore, the development of truly personalized lung cancer therapies requires further genome-specific modified cells to test drug therapies. In 2022, Naranjo developed an optimised murine lung alveolar type 2 (AT2) organoid platform that enabled realistic simulation of lung adenocarcinoma (LUAD) through multigenerational expansion (Naranjo et al., 2022). In particular, Naranjo genetically engineered organoids through genetic manipulation of AT2 cells with mutational activation of Kras, Braf, or Alk, as well as binding to the Trp53 (which corresponds to TP53 in humans) locus. In 2023, Ebisudani established 43 lines of LCOs, comprising 21 LUAD, seven SCC, 12 SCLC, and three LCNEC types (Ebisudani et al., 2023). Currently, 13 patient-derived organoid (PDO) models have been registered in clinical trials (Li et al., 2023). The lung-on-a-chip can model various lung cancer types *in situ*, similar to lung organoids. However, because of the challenging and delayed development of long-term cell culture on microchip systems, researches currently focused on *in situ* SCC, adenocarcinoma, and LCNEC. Moreover, studies on ASCs and SCLC (Yang et al., 2018; Li et al., 2021) are limited.

#### 4.1.2 Metastatic lung cancer

In addition to the aforementioned organoid and organ-on-a-chip studies on lung cancer, investigators are conducting studies on lung cancer metastasis. In 2016, Xu et al. designed a multi-organ microarray containing three layers of chambers that mimicked the invasive microenvironment of lung cancer, which can further be used to assess the mechanism of epithelial–mesenchymal transition (EMT) in lung cancer cells invading distant tissues and organs, such as the brain, bone, and liver (Xu et al., 2016). However, this lung cancer metastasis model only theorizes the impact of invasion by measuring the abnormal rate of the expression of cytokines, such as CXCR4, RANKL, and AFP to show the impact of invasion, which does not comprehensively show the interaction between lung cancer metastasis and tissues. In 2019, Nakamura et al. used a 3D organoid model containing cancer-associated fibroblasts (CAFs) and cancer cells to study the role of tumour-promoting growth factor podoplanin (+) CAFs in the proliferation and metastasis of lung adenocarcinoma cells in lung organoids. In particular, the authors evaluated the MIB-1 of podoplanin (+) and podoplanin (−) CAFs cancer cells to assess their MIB-1 index (Nakamura et al., 2019). In 2020, Xu et al. constructed a microarray model of lung cancer brain metastases (BM) to investigate the mechanism underlying BM resistance to chemotherapeutic drugs and EGFR-TKI (Xu et al., 2020). In 2021, Zheng et al. constructed a microarray model of lung cancer liver metastasis in hypoxic conditions, explored the effect of hypoxia on hypoxia-inducible factor 1α (HIF -1α), and evaluated the therapeutic effects of HIF-1α inhibitors, tirapam, SYP-5, and IDF-11774, on cancer invasion (Zheng et al., 2021). Metastasis models are frequently based on lung cancer *in situ* models, with the addition of models of other tissues outside the lung linked by inter-chip channels to simulate metastasis.

Lung organoids are suitable when the research purpose is to maintain the characteristics of the original tumour tissue as they can be easily used in tumour cells *in situ* for 3D culture and remodelling of the tumour microenvironment (TME), which is conducive to studying the mechanism of tumourigenesis and development. However, when the aim is to build a multicellular co-culture environment and observe the characteristics of tumour invasion and metastasis, lung-on-a-chips are more advantageous than lung organoids. With its unique microchannel characteristics, multiple cells can be easily co-cultured in a controlled way. Moreover, lung-on-a-chips can be used to observe tumour cells in real time, facilitated by a microscope, which enables obtaining information challenging to obtain by conventional methods.

### 4.2 Research on lung cancer related mechanisms via lung organoids and lung-on-a-chip

#### 4.2.1 Oxygen content in lung cancer

Lungs facilitate the gas exchange between the body and the outside world. The presence or absence of respiration and the level of oxygen content are crucial factors affecting tumour growth, as well as the expression of characteristics. In general, irregular growth of the vascular distance between tumour cells during the metastasis development can lead to an oxygen-rich or oxygen-suppressed state in the tumour environment (Strzyz, 2016). Hypoxia regulates the expression of molecules such as p53 (Liu et al., 2019), pyruvate kinase type M2(PKM2) (Luo et al., 2011), and HIF -1α (Gillies, 2022) in tumour cells, thus altering the glycolytic metabolism of the tumour and the expression of proteins. Jing et al. demonstrated that hypoxia can affect TME, drug resistance, DNA damage, and autophagy, leading to malignancy related to tumourigenesis (Jing et al., 2019). A similar situation was observed in lung tumours. In this regard, researchers studied lung organoids and lung-on-a-chip. In 2019, Marhuenda determined the expression levels of epithelial cell adhesion molecules (EpCAMs) in four major subtypes of lung cancer cells, H522, H1437, H1975, and H520, under different hypoxic conditions to evaluate the effects of hypoxia on the growth and invasiveness of different subtypes of lung cancer (Marhuenda et al., 2019). Jin et al., in 2019, conducted a similar study to assess whether netrin-1 regulates the migration and invasion of lung cancer cells under hypoxic conditions and explore the underlying mechanism (Jin et al., 2019). Considering that the aeration of lung organoids is closely related to membrane permeability, in 2020, Li et al. created NCI-H460X cells and compared their sensitivity to gefitinib under normoxic and hypoxic conditions. They found that hypoxia induced drug resistance in NSCLC (Li et al., 2020). In 2021, Zheng et al. designed a microfluidic chip that could precisely control the concentration of dissolved oxygen and found that under normoxic or hypoxic conditions, the HIF-1α pathway increased EMT and contributed to the metastatic progression of lung cancer to the liver (Zheng et al., 2021). On the other hand, the rate of oxygen metabolism may also reflect the extent of tumor growth. Relatedly, Nashimoto et al. have pioneered the measurement of oxygen consumption in HFL1 spheroids to further infer the rate of oxygen metabolism and assess the effects of anticancer drugs (Nashimoto et al., 2023).

#### 4.2.2 Cancer-associated fibroblasts (CAFs)

Fibroblasts in cancer tissues, also known as CAFs, are essential components of tumour stromal cells. Various studies have demonstrated that CAFs are involved in the formation of the TME by autocrine secretion of TGF-β and stromal cell-derived factor-1 (SDF-1) (Kojima et al., 2010), vascular endothelial growth factor A (VEGFA) (Guo et al., 2008), platelet-derived growth factor PDGF (Pietras et al., 2008), fibroblast growth factor (FGF), and chemokine (C-C motif) ligand 2 (CCL2) (Tsuyada et al., 2012). These cytokines or autocrine bodies are involved in stromal cell-tumour cell interactions by regulating signalling pathways such as PDGF-PDGFR and SDF-1-CXCR 4; they play crucial roles in capillary proliferation, activation, and migration of tumour cells, drug resistance generation, and immune escape during tumourigenesis (Polanska and Orimo, 2013). Similarly, novel 3D culture systems have achieved improved results in recreating CAFs in lung cancer microenvironments. In 2018, Yang et al. used co-cultured lung-on-a-chip models of A549 tumour cells and HFL1 to explore the effects of HFL1 on tumour cell metastasis and drug resistance (Yang et al., 2018). In 2019, Nakamura et al. used surgically resected lung adenocarcinoma specimens to culture a 3D organoid model of CAF-containing cancer cells. They verified the roles of tumour growth promoter CAFs and podoplanin positivity in promoting cancer cell proliferation and evaluated the MIB-1 index by comparing podoplanin (+) and podoplanin (−) cancer cells in both groups (Nakamura et al., 2019). In 2020, Chen et al. produced a 3D organoid system of SCC, co-cultured CAFs with lung squamous carcinoma cell to study the effects of CAFs on alveolar growth, morphology, and invasive destruction (Chen et al., 2020). Microarray experiments also provide a more intuitive picture of the effects of fibroblasts on lung cancer. In 2023, Monleon-Guinot et al. used transmission electron microscopy to observe and compare the morphology of two groups of organoid models: A549 mixed cultures with CAFs and A549 mixed cultures with normal fibroblasts (NF). They found that CAFs promote the upregulation of the expression levels of EMT-related genes such as CDH1 and VIM (Monleon-Guinot et al., 2023). These experimental results indicate that novel 3D models can demonstrate the complex interaction of cancer cell proliferation, invasion, and metastasis with CAFs (Kalluri and Zeisberg, 2006), providing a new platform for tumour mechanism research and drug evaluation.

#### 4.2.3 Other cues

Other cues such as respiratory movements, infection, lung fibrosis, and oncogenes play essential roles in lung cancer development. In 2017, Hassell designed a classical two-channel microfluidic microarray experiment to explore the effect of physiological respiratory exercise on the growth and invasive patterns of lung cancer, H1975 (Hassell et al., 2017). Subsequently, in 2019, Sach et al. established a respiratory syncytial virus (RSV)-infected lung organoid model, exemplifying the process of recruiting neutrophils. Moreover, they explored the interaction mechanism between cystic fibrosis (CF), RSV infection, and cancer cells (Sachs et al., 2019). The lung-on-a-chip can also demonstrate the role of normal body components in lung cancer. In 2020, Xu et al. investigated the mechanism of action (GSH) and its related enzymes, such as GPX4 and RRM2, in a BM lung-on-a-chip (Xu et al., 2020). Moreover, Surina et al. developed models of PDOs and PDSs in lung cancer and compared the effect of TGF-β in the interaction mechanism between the EMT immunity system and the stroma in both models. More importantly, oncogene-specific genetic alterations can significantly affect the development of lung cancer models. In 2022, Naranjo et al. used genetic engineering techniques to obtain LCOs with specific Kras-, Trp53-deficient, and Eml53-Alk mutations, which significantly accelerated the study of the lung cancer genetic mechanisms (Naranjo et al., 2022). In 2023, Ebisudani et al. used genetic engineering techniques to obtain NKX2-1-deficient lung adenocarcinoma carcinoid organs and discovered the mechanisms of resistance regulation in its downstream Wnt (Ebisudani et al., 2023). Currently, the combination of genetic engineering techniques and lung cancer model development is constantly evolving, facilitating the elucidation of the mechanisms of genetic factors in tumours.

The above experiments provide insights into the tumor development and resistance mechanisms based on different influencing factors. The insights enabled us to conclude that the two models, organoid and lung-on-a-chip, focus on the different tumour-influencing factors. The organoid models are more concerned with the structural study of biological tissues, such as the mechanism of CAFs and other important cellular components promoting tumour growth and invasion. In contrast, the lung-on-a-chip is focused on biological functions or physical factors affecting the cancer cells, such as the respiratory membrane of the lungs, air–liquid interface, gas shear, fluid shear, permeability of the membrane, and exchange of substances with the blood.

### 4.3 Anti-lung cancer drugs evaluation via lung organoids and lung-on-a-chip

#### 4.3.1 Chemotherapy drugs

Chemotherapy is currently the most typical clinical treatment for lung cancer. Moreover, 3D models have been used since the early days to assess the efficacy of chemotherapeutic agents. In 2015, Ying et al. investigated the effects of CAFs and paclitaxel on Met/PI78K/AKT activation and GRP3 expression using a bilayer 3D perfusion cell culture microfluidic device integrated with a concentration gradient generator (Ying et al., 2015). In 2017, Zuchowska et al. first studied 3-aminolevulinic acid (ALA-PDT) in a 3D lung cancer model using a microfluidic system (Zuchowska et al., 2017). This method can determine the safe drug concentrations and parameters for PDT. In 2019, Jung tested the response to cisplatin in SCLC (Jung et al., 2019). In 2021, Li and Zhang, using a lung organoid platform, determined that lung cancer cells were sensitised to cisplatin by inhibiting the PI3K/AKT and MAPK signalling pathways (Li et al., 2021). In addition, a combination of cisplatin and halofuginone (HF) showed that HF could sensitise lung organoids and lung cancer cells derived from patients resistant to cisplatin treatment, which may provide a new strategy to improve the prognosis of cisplatin-resistant lung cancer patients. Sankar et al., in 2021, screened the efficacy of three chemotherapeutic agents using a novel U-well 3D microfluidic chip to culture surgically resected lung cancer patient tissues. They determined that low-dose rhythmic chemotherapy could reduce the side effects of the drugs and provide better treatment outcomes (Sankar et al., 2021).

#### 4.3.2 Targeted drugs

Targeted therapeutic agents are generally based on signalling pathways or physiological processes of specific tumour genes. Common targeted therapies for lung cancer include EGFR inhibitors, such as Erlotinib and Afatinib, and ALK inhibitors, such as Crizotinib and Alectinib. In 2017, Hassell et al. investigated the efficacy of osimertinib, a third-generation epidermal growth factor receptor tyrosine kinase inhibitor (EGFR-TKI) osimertinib in a lung-on-a-chip (Hassell et al., 2017). In 2018, Yang et al. evaluated the EGFR-targeted antitumour drug gefitinib (Yang et al., 2018). Subsequently, in 2019, Kim evaluated the drug efficacy of PDO against olaparib, erlotinib, and crizotinib (Kim et al., 2019). In 2020, by constructing long-term (greater than 3 months, more than 10 generations), and short-term (less than 3 months, less than 10 generations) NSCLC organoid models, Shi found that cancer organoids with breast cancer 2 gene, EGFR, and EGFR- and EGFR-mutation/MSC-epithelial-transformation (MET)-amplified mutations responded favourably to lapatinib, erlotinib, and crizotinib, respectively (Shi et al., 2020). In the same year, Li et al. studied the role of hypoxia in inducing resistance to gefitinib using a lung-on-a-chip (Li et al., 2020). In 2022, our research team assessed the effects of the EGFR-TKIs gefitinib, osimertinib, and afatinib using a lung-on-a-chip (Tan et al., 2022). Compared with general animal models, novel 3D models for the clinical assessment of targeted drugs eliminate specificity differences between species and predict drug responses in humans more precisely (Ingber, 2022).

#### 4.3.3 Combined use of drugs

With the continuous updating of anti-lung cancer drugs, the demand for preclinical efficacy assessment combining anti-lung cancer therapies with different drugs has increased. In 2019, Sachs et al. used human airway organoids (AOs) to simulate viral infections and studied the combination of the conventional antitumour drugs paclitaxel and Nutlin-3a with crizotinib for lung cancer therapy (Sachs et al., 2019). However, there are drawbacks of fewer simulated immune cell types and less comprehensive simulation of infection in this experiment. The role of drugs combinations in the treatment of lung metastatic cancer has also been studied. In 2020, Xu et al. investigated the resistance mechanism of PC9-Br cells to chemotherapeutic agents and EGFR-TKI drugs in a BM lung-on-a-chip (Xu et al., 2020). In 2023, Wang HM conducted a LCO-based drug susceptibility test (LCO-DST) of osimertinib, paclitaxel, pemetrexed, carboplatin, etoposide, and cisplatin (Wang et al., 2023). Importantly, the study revealed the molecular mechanism of dual-target therapy by determining the proteomics of LCOs, enabling the prediction of individualised clinical responses of different patients to targeted therapy and chemotherapy.

#### 4.3.4 Other therapies

In addition, lung organoids can be used to evaluate the effects of novel antitumour drugs. In 2020, Jeong et al. designed a microfluidic lung-on-a-chip to explore the antitumour and antiangiogenic effects of miR-497 in the TME of NSCLC (Jeong et al., 2020). The study revealed that exosome-mediated miRNAs could inhibit the growth, migration, and angiogenesis of targeted tumour cells in HUVECs, providing a new clinical therapeutic option for patients with lung cancer. In the same year, Dhiman et al. used a lung-on-a-chip model to explore the potential anti-cancer effects of the tryptophan-rich peptide P1 to provide a new strategy for lung cancer treatment (Dhiman et al., 2020). In 2021, Zheng et al. explored the cancer development mechanism from lung to liver under normoxic or hypoxic conditions and drug testing of tirapamide, an HIF-1α inhibitor (Zheng et al., 2021). In 2023, Ebisudani tested C59, a novel porcupine inhibitor, for Wnt-targeted therapeutic efficacy in an NKX2-1-deficient lung adenocarcinoma chip (Ebisudani et al., 2023). These novel drug studies based on 3D culture systems provide new insights and directions for the future treatment of lung cancer.

## 5 Challenges and prospects

Lung cancer still represents a severe threat to human health despite its diagnosis and treatment progress. In particular, there is still a high risk of recurrence and a low 5-year survival rate after comprehensive treatment with surgical therapy, radiotherapy, chemotherapy, gene-targeted therapy, immunotherapy, and the implementation of various approaches. Therefore, the pathogenesis of lung cancer must be studied to develop new anti-lung cancer drugs. This review summarised various applications of advanced 3D culture systems for modelling, drug evaluation, and mechanistic lung cancer studies. However, with the continuous progress in tumour research, many researchers believe that a tumour is not just a single-organ lesion but also a systemic disease. The target organs for lung cancer metastasis not only involve the lungs but also multiple organs such as the brain, liver, adrenal glands, and bones. Thus, a single biomimetic lung-on-a-chip will not be able to simulate the entire tumour process in the human body. In addition, antitumour drugs may involve more complex pharmacokinetic reactions, such as drug absorption, metabolism, and damage to other organs. Therefore, disease and drug evaluation models are highly required. In this regard, the combination of organoids and organ-on-a-chip may represent a new direction for future research. The constantly proposed human-on-a-chip and organoid-on-a-chip is gradually developing, facilitating higher requirements for materials science, tissue engineering, artificial intelligence, *etc.* Although various challenges remain open, but more and more researchers are joining this field of research and believe that with the development of various technologies, such as micro/nanofabrication technology, intelligent materials, tissue/organ engineering, bioinformatics, *etc.*, the Advanced 3D Culture Systems are expected to solve the current research bottlenecks. In conclusion, an increasing number of studies have shown that the use of bionic organoids and organ-on-a-chip are reliable microphysiological systems that can reproduce the *in vivo* microenvironment of tumours and can be used as supplements or alternatives to 2D cultures and animal experiments.
